# Human iPSC–Derived Endothelial Cells Exhibit Reduced Immunogenicity in Comparison With Human Primary Endothelial Cells

**DOI:** 10.1155/sci/6153235

**Published:** 2024-12-09

**Authors:** Haiyan Jia, Melanie Moore, Meenu Wadhwa, Chris Burns

**Affiliations:** ^1^Biotherapeutics and Advanced Therapies, Research and Development, Science and Research Group, Medicines and Healthcare Products Regulatory Agency, Blanche Lane, South Mimms, Potters Bar EN6 3QG, Hertfordshire, UK; ^2^Therapeutic Reference Materials, Standards Lifecycle, Science and Research Group, Medicines and Healthcare Products Regulatory Agency, Blanche Lane, South Mimms, Potters Bar EN6 3QG, Hertfordshire, UK

## Abstract

Human induced pluripotent stem cell (iPSC)–derived endothelial cells (ECs) have emerged as a promising source of autologous cells with great potential to produce novel cell therapy for ischemic vascular diseases. However, their clinical application still faces numerous challenges including safety concerns such as the potential aberrant immunogenicity derived from the reprogramming process. This study investigated immunological phenotypes of iPSC-ECs by a side-by-side comparison with primary human umbilical vein ECs (HUVECs). Three types of human iPSC-ECs, NIBSC8-EC generated in house and two commercial iPSC-ECs, alongside HUVECs, were examined for surface expression of proteins of immune relevance under resting conditions and after cytokine activation. All iPSC-EC populations failed to express major histocompatibility complex (MHC) Class II on their surface following interferon-gamma (IFN-*γ*) treatment but showed similar basal and IFN-*γ*-stimulated expression levels of MHC Class I of HUVECs. Multiple iPSC-ECs also retained constitutive and tumor necrosis factor-alpha (TNF-*α*)-stimulated expression levels of intercellular adhesion molecule-1 (ICAM-1) like HUVECs. However, TNF-*α* induced a differential expression of E-selectin and vascular cell adhesion molecule-1 (VCAM-1) on iPSC-ECs. Furthermore, real-time monitoring of proliferation of human peripheral blood mononuclear cells (PBMCs) cocultured on an endothelial monolayer over 5 days showed that iPSC-ECs provoked distinct dynamics of PBMC proliferation, which was generally decreased in alloreactivity and IFN-*γ*-stimulated proliferation of PBMCs compared with HUVECs. Consistently, in the conventional mixed lymphocyte reaction (MLR), the proliferation of total CD3+ and CD4+ T cells after 5-day cocultures with multiple iPSC-EC populations was largely reduced compared to HUVECs. Last, multiple iPSC-EC cocultures secreted lower levels of proinflammatory cytokines than HUVEC cocultures. Collectively, iPSC-ECs manifested many similarities, but also some disparities with a generally weaker inflammatory immune response than primary ECs, indicating that iPSC-ECs may possibly exhibit hypoimmunogenicity corresponding with less risk of immune rejection in a transplant setting, which is important for safe and effective cell therapies.

## 1. Introduction

Vascular endothelial cells (ECs) forming the luminal surface of blood vessels display a wide array of distinct and unique physiological properties, including the control of blood flow and continued tissue perfusion, and the regulation of vascular tone and angiogenesis. However, pathological stimuli can drive endothelial dysfunction, which causes the reduction of blood supply and inadequate transport of nutrients and oxygen [1, 2]. Impaired blood circulation significantly contributes to tissue ischemia and its common clinical complications include coronary artery disease, critical limb ischemia, and stroke. Ischemic vascular disease is increasing in incidence and prevalence as populations are aging and as diabetes cases are arising. It is a major healthcare problem and existing treatment options remain limited. In particular, impaired microcirculation seriously causes tissue ischemia, and this cannot be treated by vascular surgery [3]. Therefore, alternative novel therapeutic approaches able to induce neovessel growth for replacing dysfunctional ECs and improving blood perfusion in ischemic tissues are needed to advance current treatment of the ischemic vascular disease [4–6].

Human induced pluripotent stem cell (iPSC)–derived ECs have emerged as a novel promising source of cells to promote neovascularization. iPSC-ECs showed endothelial functionality, including the ability to form capillary-like structures upon coculture and the network of perfused microvessels in vivo [7–10]. Particularly, the angiogenic potential of iPSC-ECs has been demonstrated in preclinical studies for limb ischemia, which promotes tissue regeneration and recovery of vascular function through neovascularization and vascular repair, leading to the formation of new blood vessels and increased branching of existing blood vessels [11–13]. It is, therefore, conceivable that implantation of iPSC-ECs provides great potential to produce novel cell therapy for the ischemic vascular disease and to prevent limb- or life-threatening complications. However, their clinical application still faces numerous challenges including safety concerns such as the genomic instability and potential aberrant immunogenicity derived from the reprogramming process [14–17].

Vascular ECs not only perform specialized functions in the maintenance of blood–tissue barrier and healthy vascular homeostasis, they also actively participate in immune responses [18, 19]. ECs secrete cytokines and express cytokine receptors, for example, interferon-gamma (IFN-*γ*) receptor and tumor necrosis factor-alpha (TNF-*α*) receptor. Specifically, through expression and presentation of major histocompatibility complex (MHC) molecules, Class I and Class II, on the endothelial surface, activated ECs can communicate and recruit antigen-specific CD8+ and CD4+ T cells [20–22]. In transplantation, therefore, graft ECs are both trigger and target of allogenic immune responses since they are the first allogeneic (allo) target encountered by the recipient's immune system and may display tissue-specific autoantigens in the context of an inflammatory response, causing allograft rejection. They also respond to inflammatory cytokines to interact and direct the trafficking of circulating immune cells into affected tissue through their surface expression of adhesion molecules such as E-selectin, intercellular adhesion molecule-1 (ICAM-1), and vascular cell adhesion molecule-1 (VCAM-1) [21].

While the angiogenic competence of iPSC-ECs has been well characterized, the immunological profile of iPSC-ECs is poorly defined, and the information on how closely or distantly the immune phenotype of iPSC-ECs is related to that of their native counterparts is rarely available. Especially under an inflammatory condition or allogenic environment, the antigen presentation capability and immune cell-activating potential of iPSC-ECs are not fully evaluated. In this study, we generated and characterized human iPSC-ECs and investigated the immunogenicity in vitro of multiple iPSC-EC types by examining any changes on the surface expression of MHC and adhesion molecules in response to cytokines. This was performed in a side-by-side comparison with primary human umbilical vein ECs (HUVECs), which is a well-established and most used human primary cellular model generally representing the characteristics of vascular ECs. We also compared iPSC-ECs with HUVECs in kinetic events for their ability to interact and induce the activation and proliferation of human peripheral blood mononuclear cells (PBMCs) through the real-time monitoring of PBMCs in coculture on a monolayer of ECs. Furthermore, the conventional mixed lymphocyte reaction (MLR) and cytokine release assays were employed in parallel to compare the immunogenicity properties of iPSC-ECs with HUVECs.

## 2. Materials and Methods

### 2.1. Cell Lines and Cell Culture

Human cell lines were handled in accordance with the Human Tissue Act (2004) with approval from The Human Material Advisory Committee (HuMAC) at the MHRA. NIBSC8 iPSC cell line was generated by mRNA–based reprogramming of human fibroblasts and was generously donated by the UK Stem Cell Bank. NIBSC35 was generated from human PBMCs using Sendai virus, and the production and characterization of this line has been previously published [23]. iPSCs were maintained in Essential 8 Flex Media (Life Technologies) on vitronectin (Gibco) coated 6-well plates. Media was changed every 2–3 days, and iPSCs were passaged every 4–5 days using Versene Solution (Gibco) according to the manufacturer's protocol.

Human iPSC–derived ECs, iCell ECs 01434 and iCell ECs 11713 (Catalog numbers C1021 and C1114; referred to hereafter as 01434-ECs and 11713-ECs, respectively) with the somatic origins of fibroblasts and PBMCs, respectively, were purchased from FujiFilm Irvine Scientific/Cellular Dynamics and maintained in VascuLife Growth Medium (Lifeline Cell Technology, catalog number LL-0003), according to the manufacturer's instructions, in an incubator at 37°C with 5% CO_2_. The lot-specific certificates of analysis were provided. iCell ECs at passages from 1 to 2 were used for experiments.

Primary HUVECs (Cellworks, product code: ZHC-2102) and primary human dermal fibroblasts (Cellworks, product code: ZHC-5102) were obtained from Catalog Medsystems. The lot-specific certificates of analysis were provided. HUVECs were cultured in EC growth medium-2 (EGM-2) supplemented with defined growth factors, 2% fetal bovine serum and gentamicin/amphotericin-B (Lonza Biologics Plc) in an incubator at 37°C with 5% CO_2_ and passaged by trypsinization (trypsin/EDTA solution, Lonza Biologics Plc). Fibroblasts were maintained in DMEM (Sigma D5671) supplemented with 10% fetal bovine serum and 1% L-glutamine. HUVECs and fibroblasts at passages from 3 to 8 were used for experiments.

### 2.2. Differentiation of iPSC Into EC and Maintenance

Human iPSC lines NIBSC8 and NIBSC35 were differentiated to ECs following the STEMCELL Technologies protocol. Briefly, NIBSC8 and NIBSC35 were seeded as single cells in Essential 8 Medium with the supplement (Thermo Fisher, Catalog A1517001) and 10 μM Y-27632 (STEMCELL Technologies, Catalog 72302) for 24 h. Differentiation was then initiated by replacing medium with STEMdiff Mesoderm Induction Medium (STEMCELL Technologies, Catalog 5220) for 3 days. On day 4, endothelial induction was initiated by replacing medium with STEMdiff Endothelial Induction Medium (STEMCELL Technologies, Catalog 08005) for 4 days further. On day 8, cells were harvested and expanded on cell attachment substrate-coated cultureware in STEMdiff Endothelial Expansion Medium (STEMCELL Technologies, Catalog 08007). The expansion medium was changed every 2 days for 6 days to generate ECs. These iPSC–derived ECs were passaged every 3–5 days and then cells at passages from 2 to 6 were used for experiments.

### 2.3. Cell Surface Immunostaining for Flow Cytometric Analysis

ECs or fibroblasts were harvested using trypsin–EDTA solution and collected by centrifugation. Following initial treatment with the human Fc receptor blocking reagent (Bio-Techne, 1-001-A), EC surface expression of CD31, vascular endothelial growth factor receptor 2 (VEGFR2) and vascular endothelial-cadherin (VE-cadherin) was detected using PE-conjugated anti-CD31 IgG1 monoclonal antibody (mAb; Bio-Techne, FAB3567P), PE-conjugated anti-VEGFR2 IgG1 mAb (Bio-Techne, FAB357P), and PE-conjugated anti-VE-cadherin IgG2B mAb (Bio-Techne, FAB9381P), respectively. In parallel, the cells were stained with the respective isotype-matched control mAb (Bio-Techne, IC002P or IC0041P). For validation of fibroblasts, mouse anti-human fibroblast activation protein *α* (FAP)-APC-conjugated IgG_1_ mAb (FAB3715A) and relevant isotype-matched control mAbs (Bio-Techne); and mouse anti-human fibroblasts-PE-conjugated mAb IgG_2a_ (15235909; Invitrogen MA5-16642; clone: D7-FIB) and mouse-PE-conjugated isotype-matched control IgG_2a_ (15384401; Invitrogen PA5-33207) were used.

For cell surface expression of MHC Class I and Class II molecules, PE-conjugated anti-human MHC Class I/HLA IgG_2A_ mAb (Bio-Techne, FAB7098P) and APC-conjugated anti-human MHC Class II/HLA-DR IgG_1_ mAb (Bio-Techne, FAB4869A) along with the respective isotype-matched control mAb (Bio-Techne, IC003P or IC002A) were used for staining, respectively. For cell surface expression of E-selectin, ICAM-1 and VCAM-1, PE-conjugated anti-E-selectin IgG1 mAb (Bio-Techne, FAB6169P), APC-conjugated anti-ICAM-1 IgG_1_ kappa mAb (eBioscience, 17-0549-42), and PE-conjugated anti-VCAM-1 IgG2A mAb (Bio-Techne, FAB5649P) along with the respective isotype-matched control mAb (Bio-Techne, IC002P; eBioscience, 17-4714-41; or Bio-Techne, IC003P) were used for staining, respectively. The stained cells were examined by flow cytometry using FACSCanto II (BD Biosciences) by counting 30,000 events. Data were analyzed using FACSDiva (version 6) and FlowJo (version 10) software and expressed as histogram overlayers and quantitative bar graphs with median fluorescence intensity (MFI), respectively.

### 2.4. RNA Preparation and Real-Time Quantitative PCR (qPCR)

Total RNA was isolated from cell lysates using the RNeasy Mini Kit (Qiagen, London UK) according to manufacturer's instructions. First-strand cDNA synthesis from RNA was performed using the M-MLV Reverse Transcriptase system (Promega, Southampton, UK), and the cDNA product was subject to qPCR using 2 × qPCRBIO SyGreen Blue Mix Lo-ROX (PCRBiosystem, London, UK) and 400 nM each predesigned primers (Integrated DNA technologies, Belgium). A list of primers is shown in Table 1. qPCR was performed on the Rotor-Gene Q Thermocycler, and data analyzed using Rotor-Gene Q System (QIAGEN, London, UK), 2.1.0. Gene expression was normalized to the house keeping gene *GAPDH*.

### 2.5. Real-Time Uptake of Low-Density Lipoprotein (LDL)

LDL uptake was performed using the pHrodo red dye-labelled LDL (Invitogen I34360). The pHrodo red dye-labelled LDL is dimly fluorescent at neutral pH outside of cells, but it becomes fluorescent brightly only after internalization due to its activation at acidic pH, and this property enables easy discrimination of intracellular LDL from outside and cell surface bound LDL providing greater specificity. Confluent iPSC-ECs or HUVECs in a 96-wll plate were added with the pHrodo red dye-labelled LDL in the Phenol Red-Free Opti-MEM (GIBCO, catalog number: 11058) with or without unlabeled LDL and the cell-containing plate was placed into Incucyte chamber at 37°C with 5% CO_2_ for real-time visualization and automated measurement of LDL uptake using the Incucyte Live-Cell Analysis System with the phase and orange channels and scan every 30 min over a 7-h period.

### 2.6. Real-Time Scratch Wound Cell Migration

Confluent iPSC-ECs or HUVECs in a ImageLock 96-well plate (Sartorious, catalog number 4379) were scratch-wounded using the 96-pin WoundMaker (Sartorius, catalog number 4563), and wells were washed twice with media to remove any floating cells. Immediately following wounding, the cells were treated without or with VEGF (Bio-Techne, 293-VE) at 25 ng/ml in Medium 199 (Sigma–Aldrich, catalog number M4530) supplemented with 2% fetal bovine serum and the cell-containing ImageLock plate was placed into Incucyte chamber at 37°C with 5% CO_2_ for real-time visualization and automated measurement of cell migration into the wound area using the Incucyte Live-Cell Analysis System with the phase contrast channel, Scratch Wound Module (Sartorius catalog number 9600-0012) and scan every 1 h over a 24-h period.

### 2.7. Coculture Model of Angiogenesis

Angiogenesis was evaluated in a coculture assay of primary HUVECs and primary human fibroblasts in a 24-well plate format by a newly developed stepwise seeding approach to replace a coseeding of both HUVECs and fibroblasts as previously described [24, 25]. Briefly, fibroblasts were seeded in 24-well plates in DMEM (Sigma D5671) supplemented with 10% fetal bovine serum and 1% L-glutamine for growth to create a monolayer as the first step, and then, HUVECs were seeded on the monolayer of fibroblasts in EC Basal Medium (Lonza catalog number CC3121) supplemented with 2% fetal bovine serum at day 1. VEGF (Bio-Techne, 293-VE) at 10 ng/ml alone or in combination with bevacizumab (Bev; Roche) at 5 µg/ml was administered to the coculture at day 2 and at each medium change. At day 9, the cultures were fixed and immunostained for the EC marker CD31 using mouse anti-human CD31 primary antibody (Bio-Rad, MCA1738), followed by goat anti-mouse IgG secondary antibody conjugated with alkaline phosphatase (Bio-Rad catalog number STAR132a) and the enzyme substrate of 5-bromo-4-chloro-3-indolyl phosphate/nitro blue tetrazolium solution (Sigma–Aldrich, B5655-25TAB). The image of the stained EC–derived tubules was photographed using Olympus inverted microscope with ×4 objective lens and Nikon imaging system. Angiogenesis images (eight images from duplicate wells per condition) were quantitatively analyzed using the AngioSys image analysis software (Cellworks).

### 2.8. Isolation of Human PBMCs

Human PBMCs were isolated using Histopaque-1077 density separation from leucocyte cones obtained from the National Health Service Blood and Transfusion, kindly performed by Anna Nowocin. Isolated PBMCs were then stored frozen over liquid Nitrogen as stocks of 1–5 × 10^7^ cells until required. PBMC were thawed and rested overnight at a density of 2 × 10^6^ cells per ml complete RPMI, (RPMI 1640 supplemented with 10% FCS, 2 mM L-glutamine, and 100 U/ml penicillin and 100 µg/ml streptomycin (all Gibco)).

### 2.9. Real-Time Proliferation of PBMCs Cocultured on a Monolayer of ECs

Human PBMCs as responder cells were plated at a seeding density of 10,000 cells per well on a monolayer of iPSC-ECs or HUVECs as stimulated cells in a 96-well plate and the cocultures were treated without or with IFN-*γ* (Bio-Techne, catalog number 285IF) at 10 ng/ml in EGM (Lonza, catalog number CC3124). PBMCs were also seeded at the density of 10,000 cells per well on 0.01% poly-L-ornithine solution (Sigma–Aldrich, Catalog number P4957) coated wells in the absence of ECs to serve as EC-free untreated control. The cell-containing plate was placed into Incucyte chamber at 37°C with 5% CO_2_ for real-time visualization and automated measurement of PBMC proliferation using the Incucyte Live-Cell Analysis System with the phase contrast channel and nonadherent cell-by-cell scan every 1 h over a 5-day period.

### 2.10. One-Way MLR and Cytokine Release

To compare the immunogenic potential of iPSC–derived EC and HUVEC, a one-way MLR assay was used with PBMC as the responder cells and NIBSC8–derived EC (referred to hereafter as N8-EC), 11713-EC, 01434-EC, or allo PBMC as stimulator cells. 2 × 10^5^ responder cells were cocultured with 1 × 10^4^ stimulator cells and maintained in complete EGM media in 96-well plates at 37°C in 5% CO_2_ for 5 days. To assess T-cell proliferation of responders, cells were labeled with fixable viability dye (FVD), T cell markers (all BD Biosciences) CD3 (558117), CD4 (557871), and CD8 (560662), and proliferation marker anti-Ki67 (561283). Live single cells were gated for each fluorophore using fluorescence minus one controls. The expression of the human Ki67 protein is strictly associated with cell proliferation [26]; and therefore, Ki67+ cells denote proliferating cells. Levels of proliferation were numerically expressed as stimulation index (SI), calculated as the number of Ki67+ cells of each one-way MLR over the number of Ki67+ cells in a negative (autologous PBMC only) control [27, 28]. As a positive control for T cell activation, PBMCs were stimulated with Dynabeads Human T-Activator CD3/CD28 (Gibco) according to the manufacturer's instructions.

Cytokine concentrations from the supernatants of 5-day cocultures were measured using the V-PLEX human cytokine kit (Mesoscale) detecting IFN-*γ*, IL-2, IL-6, IL-8, IL-10, IL-12p70, and TNF-*α*. Supernatants were diluted 1:10 into the manufacturers provided assay diluent, plates prepared according to the manufacturer's protocol, and the signal detected by the MESO Quick Plex SQ 120. Data analysis was performed using the MSD DISCOVERY WORKBENCH analysis software.

### 2.11. Statistical Analysis

Data were analyzed using GraphPad Prism (version 10) statistical packages. Differences between two groups were assessed using the unpaired *t* test or unpaired *t* test with Welch's correction where appropriate. Differences among three or four groups were assessed using the one-way analysis of variance (ANOVA) with Bonferroni's multiple comparison tests or mixed effects model with Dunnetts multiple comparison tests. Bar graphs represent means ± SEM determined from the results of three independent experiments each performed in two–four replicates unless where stated. A value of *p* < 0.05 was taken as statistically significant.

## 3. Results

### 3.1. Differentiation of iPSCs to ECs

**Figure 1:**
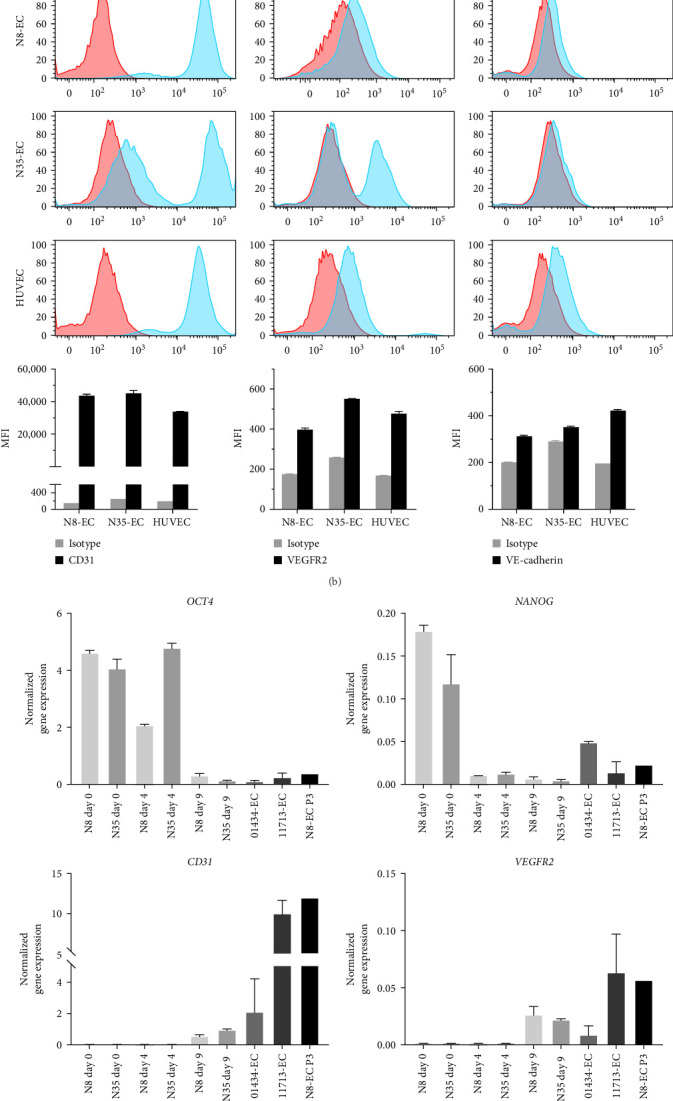
Generation and molecular characterization of iPSC-ECs. (a) Schematic representation of differentiation stages and phase-contrast images of endothelial generation from two iPSC lines NIBSC8 and NIBSC35. Both NIBSC8 and NIBSC35 were pluripotent at day 0 and went through mesoderm induction and endothelial differentiation up to day 8. On day 14, iPSC-ECs displayed a characteristic EC cobble-stone morphology. (b) Representative histogram overlays and quantitative bar graphs showed flow cytometric analysis of surface expression of CD31, VEGFR2, and VE-cadherin using specific fluorescence-labelled antibodies alongside isotype-matched antibodies on iPSC-ECs and HUVECs, respectively. Pink histograms represent isotype-matched control staining and blue histograms represent specific antibodies for indicated molecules staining. (c) qPCR was performed on cDNA prepared from NIBSC8 and NIBSC35 at day 0, 4, and 9 of endothelial differentiation for pluripotency markers OCT4 and NANOG and key EC markers CD31, VEGFR2, VE-cadherin, and vWF (*n* = 3 per cell line). All EC markers were upregulated, and pluripotency markers downregulated by day 9 of differentiation compared to the pluripotent day 0 control. Results were normalized to GAPDH. cDNA prepared from two commercial iPSC-EC lines 01434-EC and 11713-EC (*n* = 2 per cell line) and N8-EC at passage 3 (*n* = 1) were included for comparison. 01434-EC, iCell ECs 01434; 11713-EC, iCell ECs 11713; EC, endothelial cell; HUVECs, human umbilical vein ECs; iPSC, induced pluripotent stem cell; MFI, median fluorescence intensity; qPCR, quantitative PCR; VE-cadherin, vascular endothelial-cadherin; VEGFR2, VE growth factor receptor 2; vWF, von Willebrand factor.

We utilized two in-house human iPSC lines, NIBSC8 and NIBSC35, originating from fibroblasts and PBMCs, respectively [23], for differentiation into ECs by employing the Stemcell Technologies' monolayer culture-based protocol. It was a stepwise differentiation of iPSCs through mesoderm induction to endothelial differentiation using the STEMdiff Mesoderm Induction Medium and Endothelial Differentiation Kit. On day 14, the differentiated cells showed the characteristic EC cobblestone morphology (Figure 1a). The endothelial phenotype of the differentiated cells was characterized by detecting the expression of EC surface markers such as CD31, VEGFR2, and VE-cadherin using flow cytometry. Compared with isotype controls, NIBSC8-derived ECs expressed CD31, VEGFR2, and VE-cadherin, which showed similar expression levels to those on the surface of HUVECs (Figure 1b), suggesting that they were well differentiated. However, only a subset of NIBSC35–derived ECs (referred to hereafter as N35-ECs) expressed CD31 and VEGFR2 alongside a weak expression of VE-cadherin compared with those on HUVECs (Figure 1b).

qPCR was performed to assess the levels of gene expression associated with ECs as they differentiated. Across the differentiation period, for both NIBSC8 and NIBSC35, expression of the pluripotent cell markers OCT4 and NANOG both deceased by day 9 of differentiation, with a corresponding increase in the endothelial markers CD31, VEGFR2, VE-cadherin, and von Willebrand factor (vWF; Figure 1c). cDNA obtained from two commercial human iPSC-ECs, 01434-ECs and 11713-ECs, and the N8-ECs after 3 passages, were also analyzed for their expression of pluripotent associated and EC associated markers. As described above, the N35-EC was not cultured further due to the lower expression of CD31, VEGFR2, and VE-cadherin expression observed by flow cytometry. Whilst the expression of the EC associated genes in N8-EC and N35-EC were comparable at day 9 to one of the commercial iPSC-EC lines, 01434-EC, they were noticeably lower than the expression of these markers in the 11713-EC line. However, the N8-EC line after passage showed a considerable increase in the expression of all the EC associated genes, which was also comparable to that of the 11713-EC commercial line (Figure 1c).

The data demonstrates some variability in the expression of EC markers across the iPSC derived EC lines, and that this may reflect variability in the differentiation efficiency or maturation level of these lines. The data also indicates there was further maturation toward the endothelial lineage as the N8-EC line was cultured.

As N8-ECs display similar lineage marker expression to primary HUVECs and represent EC identity, they were, therefore, subjected to further functional characterization.

### 3.2. Functional Characterization of iPSC-ECs

**Figure 2:**
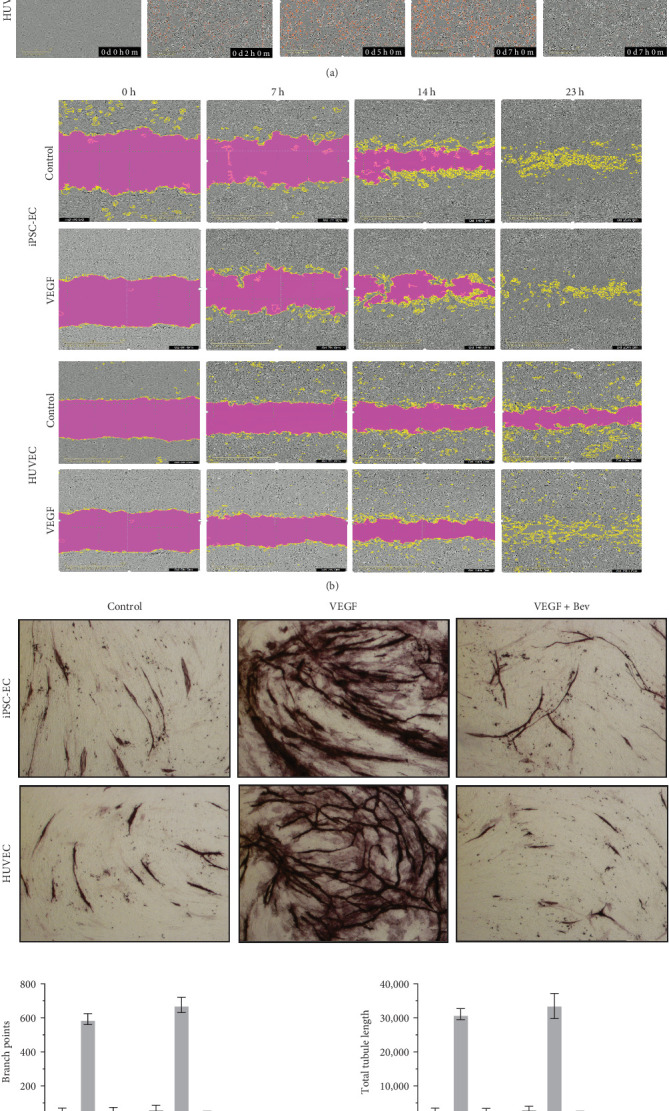
Biological functionality of iPSC-ECs. (a) Time-lapse images for the phase object and orange object showed an increase in LDL uptake by both iPSC-ECs and HUVECs at different time points over the 7-h time course and this was blocked specifically by unlabeled LDL monitored by the Incucyte Live-Cell Analysis System. (b) Time-lapse phase-contrast images showed cell migratory movements of untreated and VEGF-treated iPSC-ECs or HUVECs to the wound area at different time points over the 23-h time course monitored by the Incucyte Live-Cell Analysis System. (c) iPSC-ECs or HUVECs were seeded on a monolayer of fibroblasts and were cocultured over a period of 9 days. The coculture was treated without or with VEGF at 10 ng/ml in the absence or presence of 5 µg/ml Bev at day 2 and at each medium change. Brightfield images of EC–derived microtubules were visualized by immunostaining for CD31 at day 9. Quantification of tubule images (eight images from duplicate wells per condition) was shown as number of branch points and total tubule length. See also Supporting Information 1: Figure S1 for validation of human primary fibroblasts, Supporting Information 1: Videos S1 and S2 for LDL uptake and Supporting Information 1: Videos S3–S6 for wound scratch cell migration in the Supporting Information. Bev, bevacizumab; ECs, endothelial cells; HUVECs, human umbilical vein ECs; iPSC, induced pluripotent stem cell.

Next, we investigated whether iPSC-ECs were functionally similar to primary HUVECs. Uptake of LDL is a functional assay for ECs, which express specific LDL receptor for LDL endocytosis. By monitoring a kinetic uptake of the pHrodo red dye-labelled LDL (Invitogen) in live ECs using the Incucyte Live-Cell Analysis System, we observed an increase in LDL uptake by HUVECs over a 7-h time course (Figure 2a and Supporting Information 1: Video S1). This endocytosis was blocked by unlabeled LDL, reflecting the specific LDL receptor–mediated endocytosis. Like HUVECs, iPSC-ECs were also able to uptake LDL specifically (Figure 2a and Supporting Information 1: Video S2).

As EC migration is a critical, initial step in the angiogenic process, the Incucyte real-time measurement of scratch wound cell migration was employed to compare iPSC-ECs with HUVECs. Figure 2b showed that VEGF-treated HUVECs migrated fast into the wound region and made a closure of the wound, whereas the wound region was not closed in the absence of VEGF over a period of 23 h (Supporting Information 1: Videos S3 and S4). The migration-related wound closure of iPSC-ECs was also rapid in the presence of VEGF compared with the untreated control (Figure 2b and Supporting Information 1: Videos S5 and S6).

To further characterize the angiogenic potential of iPSC-ECs, we first validated the purity of human primary fibroblasts (Supporting Information 1: Figure S1) and then established a stepwise coculture by adding iPSC-ECs on a monolayer of fibroblasts to develop the capillary-like structures in vitro, recapitulating physiological microvessel formation. As expected, exposure of the coculture of HUVECs with fibroblasts to VEGF at day 9 enhanced the development of EC–derived capillary-like network with an increase in the density and total length of microtubules and branch points compared with the untreated control as evidenced by immunostaining with CD31, and this was specifically inhibited by Bev, an anti-VEGFmAb (Figure 2c). Importantly, iPSC-ECs responded to VEGF stimulation and formed a similar capillary-like structure to that presented by HUVECs, which was again specifically inhibited by Bev (Figure 2c). The angiogenic potential of iPSC-ECs is consistent with their ability to migrate and close the wound area.

Taken together, these data from biological characterization confirmed that ECs derived from iPSCs manifested the endothelial identity, maturity, and functionality, suggesting that the cells could be applied for following immunological assessment.

### 3.3. Comparison of Surface Expression of MHC Molecules Between iPSC-ECs and HUVECs

**Figure 3:**
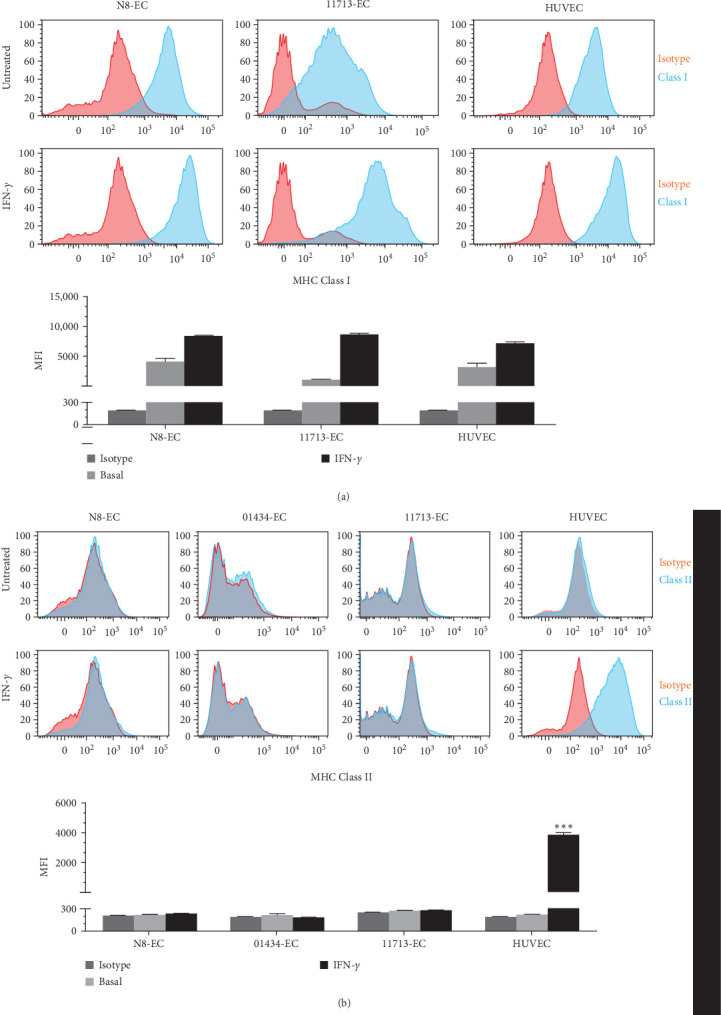
MHC molecule phenotype and responsiveness of iPSC-ECs in comparison with HUVECs. The iPSC-ECs and HUVECs were treated in the absence and presence of IFN-*γ* at 10 ng/ml for 24 h. The cell surface expression of MHC Class I (a) and MHC Class II (b) was detected by flow cytometry using specific fluorescence-labelled antibodies alongside isotype-matched antibodies, respectively. Pink histograms represent isotype-matched control staining, and blue histograms represent specific antibodies for MHC molecules staining. *⁣*^*∗∗∗*^*p* < 0.001 for IFN-*γ*-treated HUVECs versus basal untreated control. ECs, endothelial cells; HUVECs, human umbilical vein ECs; IFN-*γ*, interferon-gamma; iPSC, induced pluripotent stem cell; MFI, median fluorescence intensity; MHC, major histocompatibility complex.

Having demonstrated the endothelial functions of iPSC-ECs, we proceeded to address the major research question of this study, that is, the immunogenicity profile of iPSC-ECs in comparison with primary ECs including the response to inflammatory stimuli and participation in interaction and activation of immune cells. N8-ECs together with two commercial types of human iPSC-ECs, 01434-ECs and 11713-ECs, were first analyzed for their surface expression of MHC Class I and Class II, the crucial mediators of immunogenicity. iPSC-EC alongside HUVECs were evaluated under basal conditions and after IFN-*γ* activation. In agreement with previous reports [29, 30], MHC class I was present on the surface of resting HUVECs, and it was further increased by IFN-*γ* stimulation to a high level (Figure 3a). Both N8-ECs and 11713-ECs mirrored the basal and IFN-*γ*-activated expression levels of MHC Class I of HUVECs (Figure 3a). Resting HUVECs did not present MHC Class II on their surface, but were induced to express MHC Class II by IFN-*γ* treatment as expected (Figure 3b). Like HUVECs, resting N8-ECs, 01434-ECs and 11713-ECs did not constitutively express MHC Class II. However, unlike HUVECs, all three types of iPSC-ECs were unable to respond to IFN-*γ* stimulation for the induction of surface expression of MHC class II (Figure 3b), indicating the disparity in IFN-*γ*-induced inflammatory response relevant for communicating with the antigen-specific CD4+ T cells between iPSC-ECs and primary ECs.

### 3.4. Comparison of Surface Expression of Adhesion Molecules Between iPSC-ECs and HUVECs

**Figure 4:**
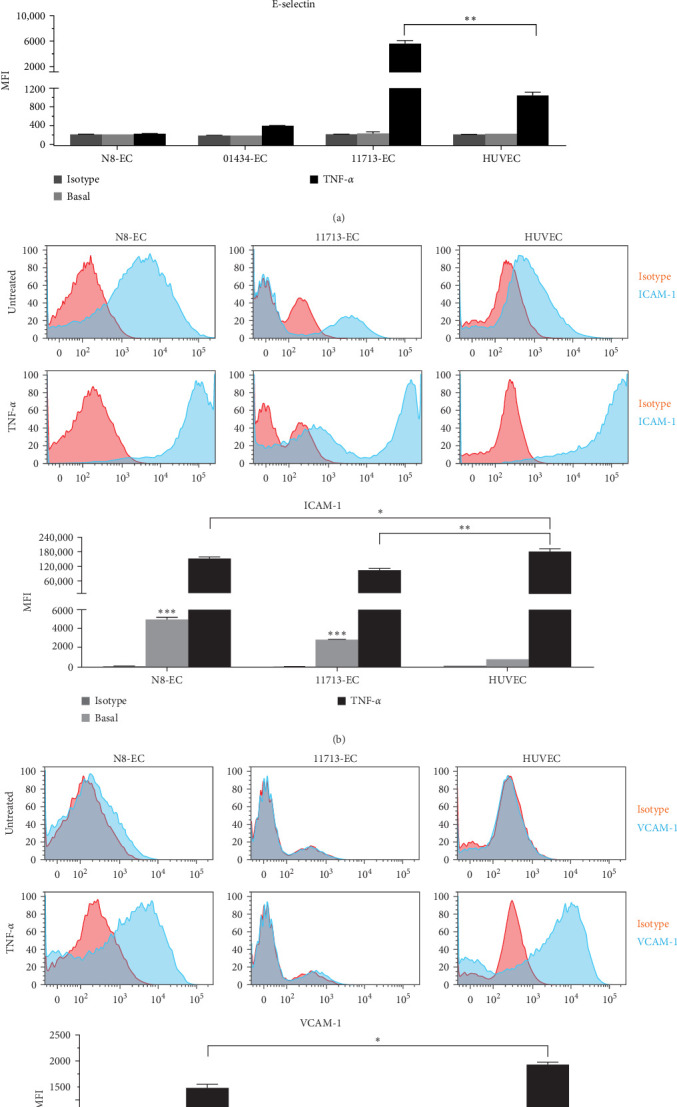
Adhesion molecule phenotype and responsiveness of iPSC-ECs in comparison with HUVECs. The iPSC-ECs and HUVECs were treated in the absence and presence of TNF-*α* at 10 ng/ml for 24 h. The cell surface expression of E-selectin (a), ICAM-1 (b), and VCAM-1 (c) was detected by flow cytometry using specific fluorescence-labelled antibodies alongside isotype-matched antibodies, respectively. Pink histograms represent isotype-matched control staining, and blue histograms represent specific antibodies for adhesion molecules staining. *⁣*^*∗∗*^*p*  < 0.01 for TNF-*α*-treated 11713-ECs versus TNF-*α*-treated HUVECs; *⁣*^*∗∗∗*^*p* < 0.001 for untreated N8-ECs or untreated 11713-ECs versus untreated HUVECs; *⁣*^*∗*^*p*  < 0.05 for TNF-*α*-treated N8-ECs versus TNF-*α*-treated HUVECs. ECs, endothelial cells; HUVECs, human umbilical vein ECs; ICAM-1, intercellular adhesion molecule-1; iPSC, induced pluripotent stem cell; MFI, median fluorescence intensity; N8-ECs, NIBSC8–derived ECs; TNF-*α*, tumor necrosis factor-alpha; VCAM-1, vascular cell adhesion molecule-1.

We next investigated the adhesion molecule phenotypes of iPSC-ECs versus those of HUVECs for the surface expression of E-selectin, ICAM-1, and VCAM-1 required for the interaction and recruitment of immune cells. As shown in Figure 4a, E-selectin was hardly detectable on the surface of resting HUVECs, and it was induced following TNF-*α* stimulation, which is consistent with previous studies [31]. Interestingly, we observed a differential expression of TNF-*α*-induced E-selectin among iPSC-EC types, either negative on N8-ECs or a fraction on 01434-ECs or a significantly higher level on 11713-ECs than that on HUVECs, while these resting iPSC-ECs resembled HUVECs with the lack of constitutive expression of E-selectin (Figure 4a). Both resting iPSC-ECs and HUVECs expressed ICAM-1, which was enhanced to a high level following exposure to TNF-*α* with a lesser extent in iPSC-ECs than HUVECs (Figure 4b). It was noticed that compared to HUVECs, both N8-ECs and 11713-ECs possessed a significant high level of basal expression of ICAM-1, and 11713-ECs exhibited a different expression pattern of ICAM-1. Furthermore, VCAM-1 was not present on the surface of resting HUVECs, and it was induced by TNF-*α* treatment. N8-ECs and 11713-ECs, like HUVECs, had no constitutive expression of VCAM-1. Contrary to HUVECs, 11713-ECs were not induced to express VCAM-1 after TNF-*α* activation (Figure 4c). In addition, N8-ECs showed TNF-*α*-stimulated expression levels of VCAM-1, but to a lesser degree than HUVECs (Figure 4c). These findings of differential expression of TNF-*α*-induced E-selectin and VCAM-1 suggest the heterogeneous inflammatory responses across different types of iPSC-ECs.

### 3.5. Comparison of Induction of Alloreactivity and Inflammatory Response of PBMCs Between iPSC-ECs and HUVECs

Considering the observed variability in cytokine-induced expression of MHC Class II, E-selectin, and VCAM-1 between iPSC-ECs and HUVECs, we then asked what their functional impacts could be in terms of initiating inflammatory immune responses such as immune cell proliferation. To gain dynamic insights into the phenotypic and functional changes in immune cells in interacting with iPSC-ECs or primary ECs and their responses to cytokines, we developed a new coculture model for real-time measurement of proliferation of PBMCs (responders) in direct contact with iPSC-ECs or HUVECs (stimulators) using the Incucyte Live-Cell Analysis System. This model proved to capture kinetic events surrounding the activation and proliferation of PBMCs on a monolayer of ECs.

**Figure 5:**
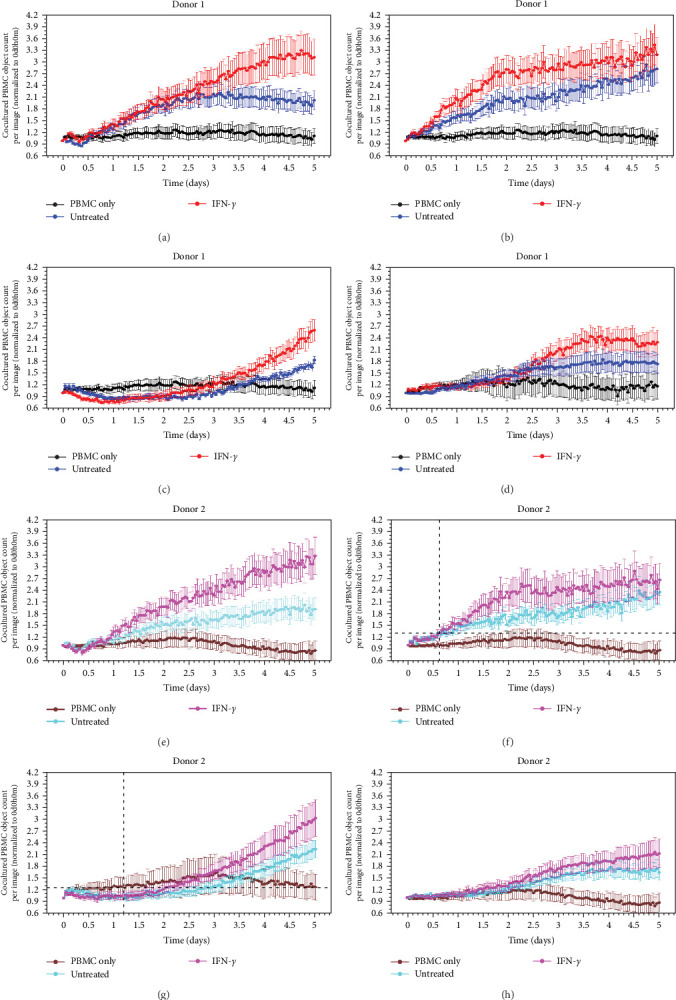
Differentially dynamic changes in the proliferation of PBMCs in coculture with iPSC-ECs or HUVECs over time. Real-time monitoring of proliferation of mismatched PBMCs from D1 (a–d) and D2 (e–h) as responder cells in coculture on a monolayer of HUVECs (a, e), N8-ECs (b, f), 11713-ECs (c, g), and 01434-ECs (d, h) as stimulator cells in the absence or presence of IFN-*γ* at 10 ng/ml over a period of 5 days was quantified using the Incucyte Non-Adherent Cell-by-Cell Analysis software for segmenting phase-contrast images. PBMC only was used as EC-free untreated control. Values shown are as the mean ± SD of images from four wells per condition. See also Supporting Information2: Videos S7–S10. 01434-ECs, iCell ECs 01434; 11713-ECs, iCell ECs 11713; D1, donor 1; D2, donor 2; ECs, endothelial cells; HUVECs, human umbilical vein ECs; IFN-*γ*, interferon-gamma; iPSC, induced pluripotent stem cell; N8-ECs, NIBSC8–derived ECs; PBMC, peripheral blood mononuclear cells.

As shown in Figure 5a,e, PBMCs (donor 1 (D1) and donor 2 (D2)) by themselves failed to proliferate over a 5-day period course. Upon coculturing on a monolayer of HUVECs, these mismatched PBMCs were able to proliferate in a time-dependent manner, suggesting primary EC–induced PBMC allogenic response. Exposure of the coculture to IFN-*γ* caused a further continuous increase in the proliferation of PBMCs on the HUVEC monolayer (Supporting Information 2: Videos S7 and S8). The PBMCs in coculture on a monolayer of N8-ECs also showed an increase in growth during the time course compared to the PBMCs only control, and they responded to IFN-*γ* treatment but reached a plateau after 2 days (Figure 5b,f and Supporting Information 2: Videos S9 and S10), and the inflammatory responses were slightly reduced to those of PBMCs cocultured with HUVECs. Interestingly, there was little basal and IFN-*γ*-stimulated proliferation of PBMCs when cocultured on a monolayer of 11713-ECs during the first 2 days and the growth of PBMC in the presence of IFN-*γ* on the 11713-ECs monolayer was comparably lower than that of PBMCs cocultured with HUVECs at the end of time course (Figure 5c,g). Similarly, these PBMCs on a monolayer of 01434-ECs showed no response during the first 2 days and a weak stagnant growth after 3.5 days in response to IFN-*γ* (Figure 5d,h). It was interesting to observe different growth dynamics (either delayed or stagnant) of PBMCs in coculture on the iPSC-EC monolayer as compared with a continuous proliferation of PBMCs on the HUVEC monolayer under inflammatory environments.

The data showed that PBMCs from two different donors in coculture with the three types of iPSC-ECs exhibited a similar pattern in alloreactive and IFN-*γ*-stimulated proliferation, and it was attenuated when compared to those on the HUVEC monolayer. Collectively, these findings indicate that iPSC-ECs elicit a weaker alloreactive and inflammatory response in PBMCs than primary ECs.

### 3.6. Comparison of iPSC-ECs With HUVECs in the Induction of Alloreactivity of T Cells and Cytokine Release

**Figure 6:**
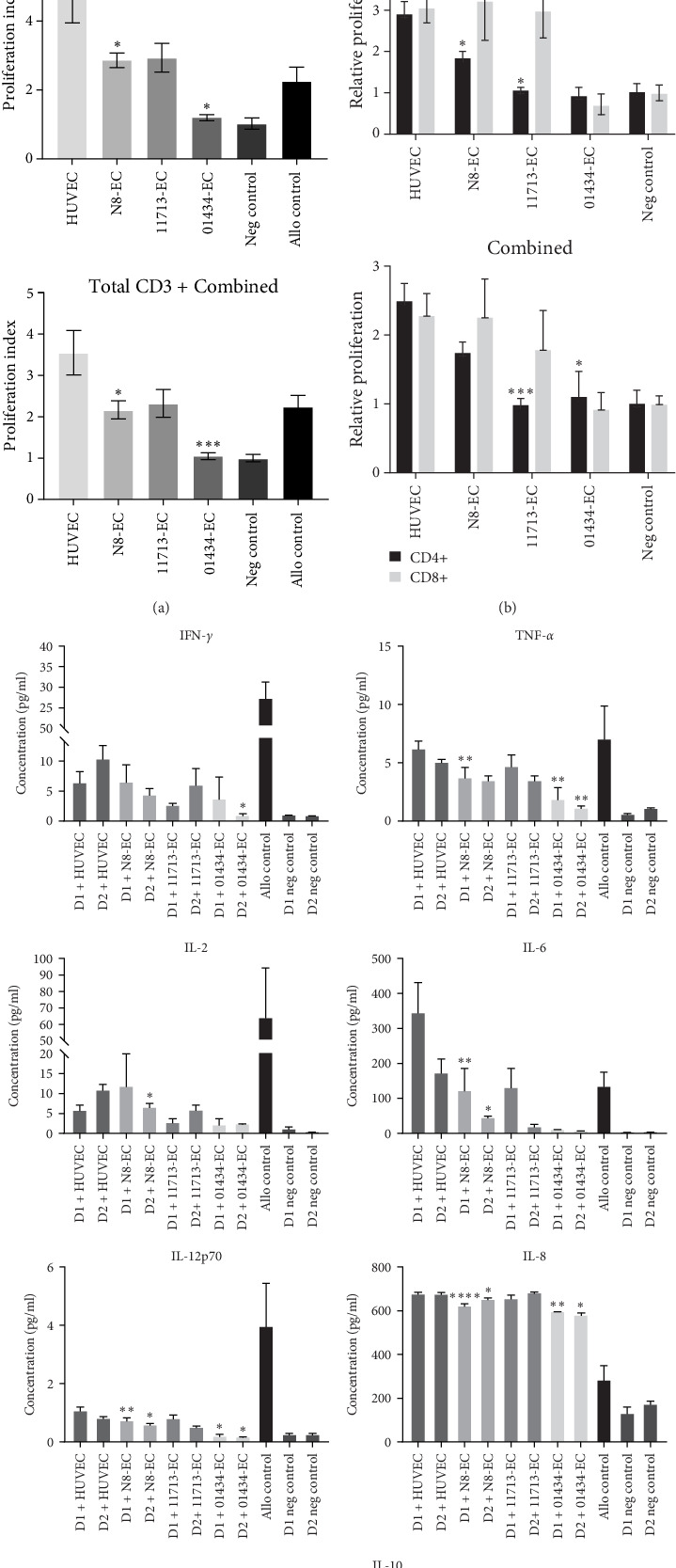
Alloresponse potential of T cells in co-culture with iPSC-ECs or HUVECs. (a) Total CD3+ proliferation of responder PBMC alone (Neg control) and after coculture with allo PBMC, HUVEC, N8-EC, 11713-EC, and 01434-EC for 5 days. Proliferation of each individual donor, and the donors combined is shown. Proliferation was measured by Ki67 staining of CD3+ T cells by flow cytometry and is shown here as proliferation index (total CD3+ proliferation in coculture/neg control). *n* = 3 separate experiments for 11713-EC, *n* = 2 for N8-EC, and *n* = 1 for 01434-EC, each sample in quadruplicate or triplicate per experiment. (b) CD3+, CD4+, and Ki67+ and CD3+, CD8+, and Ki67+ subpopulations of responder PBMC (D1 and D2, respectively, and combined) after coculture with N8-EC, 11713-EC, and 01434-EC. Data is shown here as proliferation relative to that in the neg control. *n* = 2 separate experiments for 11713-EC and N8-EC and *n* = 1 for 01434-EC, each sample in quadruplicate or triplicate per experiment. (c) Cytokines released into the supernatants of cocultures from (b) above, with allo PBMC, HUVEC, N8-EC, 11713-EC, and 01434-EC for 5 days were measured using a multiplex immunoassay (proinflammatory 10-plex, Mesoscale Diagnostics). Supernatants from the 5-day cocultures were assayed for IFN-*γ*, IL-2, TNF-*α*, IL-10, IL-12p70, IL-6, and IL-8. *n* = 2 separate experiments for 11713-EC and N8-EC and *n* = 1 for 01434-EC, each sample in quadruplicate or triplicate per experiment. Statistical analysis was performed by mixed effects model with Dunnetts multiple comparison tests. Asterisks denote statistical significance in comparison to the HUVEC-PBMC control: *⁣*^*∗*^*p* ≤ 0.05, *⁣*^*∗∗*^*p* ≤ 0.01, *⁣*^*∗∗∗*^*p* ≤ 0.001. 01434-EC, iCell ECs 01434; 11713-ECs, iCell ECs 11713; Allo, allogeneic; D1, donor 1; D2, donor 2; ECs, endothelial cells; HUVECs, human umbilical vein ECs; IFN-*γ*, interferon-gamma; iPSC, induced pluripotent stem cell; N8-EC, NIBSC8–derived ECs; Neg, negative; PBMC, peripheral blood mononuclear cell; TNF-*α*, tumor necrosis factor-alpha.

In parallel with the real-time monitoring of PBMC proliferation experiments, we investigated whether iPSC-EC coculture could modulate the alloantigen-reactivity of T cells using a conventional end point one-way MLR assay, as alloreactive T cells are central mediators of allograft rejection. PBMC were removed after 5 days coculture and stained for T cell markers and the proliferation marker Ki67 (Figure 6). The proliferation index was calculated as the percentage T cell proliferation over that seen in the negative (autologous) PBMC control. Similar to the kinetic assays, there was some donor variability in the level of PBMC response observed to the EC populations. In D1, HUVEC gave a total CD3+ proliferation index of 1.8 ± 0.15 compared with 1.4 ± 0.14 for N8-EC, 1.5 ± 0.33 for 11713-EC, and 0.9 ± 0.05 for 01434-EC and in D2, HUVEC gave a proliferation index of 4.7 ± 0.82 compared with 2.85 ± 0.22 for N8-EC, 2.91 ± 0.42 for 11713-EC, and 1.2 ± 0.10 for 01434-EC (Figure 6a). The data indicates that proliferation of total CD3+ T cells was reduced in all iPSC-EC populations compared to HUVEC, although only to a significant level in N8-EC and 01434-EC.

The CD4+ and CD8+ T cell subpopulations were also stained with Ki67, and proliferation is shown here in Figure 6b as relative proliferation over that seen in the negative (autologous) control. In comparison with the HUVEC cocultures, there is an overall trend with both PBMC donors for reduced CD4+ T cell proliferation in the cocultures with iPSC-EC populations, which is significant for N8-EC and 11713-EC in D2. However, when the donor responses were combined (Figure 6b), reduced CD4+ T cell proliferation was significant with 11713-EC and 01434-EC populations. With the exception of reduced proliferation in 11713-EC with D1 and 01434-EC with D2 (although this was not to statistical significance), this trend was not observed with CD8+ T cell proliferation.

To provide supporting information regarding the inflammatory response to iPSC-EC in the end point assay, the concentrations of proinflammatory cytokines were measured in the supernatants obtained at the end of the 5-day cocultures. Figure 6c shows the concentrations of IFN-*γ*, TNF-*α*, IL-2, IL-6, IL-12p70, and IL-8 in cocultures of HUVEC and the iPSC-EC populations with both D1 and D2. The anti-inflammatory cytokine, IL-10, was also included in the panel. Reflecting the proliferation response observed in the PBMC from these cocultures, there is a general trend across the array of inflammatory cytokines measured for reduced concentration in the iPSC-EC cocultures compared to the HUVEC cocultures, and that this is significantly different in the N8-EC and 01434-EC for TNF-*α*, IL-6, IL-12p70, and IL-8. For IFN-*γ* and IL-2, levels are more variable between the donors, reflecting that seen in the proliferation responses above, but in general, iPSC-EC cocultures have lower concentration of these cytokines compared to HUVECs. For example, for D1, IFN-*γ* concentrations were 6.4 pg/ml in HUVEC cocultures compared to 6.5 pg/ml in N8-EC, 2.5 pg/ml in 11713-EC, and 3.8 pg/ml in 01434-EC; whereas for D2, IFN-*γ* concentrations were 10.5 pg/ml in HUVEC compared with 4.3 pg/ml in N8-EC, 5.9 pg/ml in 11713-EC, and 0.7 pg/ml in 01434-EC co-cultures.

Although it is not known which cell type is responsible for the cytokine production in these cocultures, it is interesting to note the large differences in levels of IL-6 and IL-8 in cocultures compared to the allogenic control, indicating there may be some production of these cytokines from the EC populations in response to inflammation, but to a lesser extent in the iPSC-ECs compared with HUVECS. For example, in D1 + HUVEC cocultures, IL-6 concentration was 350 pg/ml compared with 125 pg/ml in N8-EC, 132 pg/ml in 11713-EC, and 10.2 pg/ml in 01434-EC, and IL-8 concentration in D1 cocultures was 674 pg/ml in HUVEC compared with 619 pg/ml in N8-EC, 653 pg/ml in 11713-EC, and 594 pg/ml in 01434-EC.

IL-10 is a well-known anti-inflammatory cytokine, and although at odds to the overall inflammatory response observed, IL-10 concentrations were also increased in the HUVEC coculture supernatants compared with iPSC-ECs (Figure 6c). Again, as it is not known which cell type is involved in the cytokine production of IL-10 levels in these cocultures, and at this time point, may be more generally reflective of the immune response.

## 4. Discussion

In this work, we produced human iPSC-ECs with similar endothelial phenotypic marker expression and biological function to those of primary HUVECs. By the side-by-side comparison with primary HUVECs, we assessed the immune phenotype, inflammatory response, and allogenic antigenicity across multiple types of iPSC-ECs to address their immunogenic status. We showed that iPSC-ECs were largely indistinguishable from HUVECs in their constitutive expression of MHC Class I, and their response to IFN-*γ* upregulation of this molecule. Both iPSC-ECs and HUVECs had no basal expression of MHC Class II, but all iPSC-EC populations differed from HUVECs with a lack of expression of MHC Class II in response to IFN-*γ* induction. Furthermore, iPSCs were very similar to HUVECs in the absence of constitutive expression of both E-selectin and VCAM-1, and presence of basal expression and TNF-*α*-stimulated expression levels of ICAM-1. However, they manifested a mixed reaction to TNF-*α* induction of E-selectin and VCAM-1. Functionally, iPSC-ECs elicited different proliferation dynamics of PBMCs, generally being less able to induce alloreactive and IFN-*γ*-stimulated proliferation of PBMCs than HUVECs during 5-day cocultures. Consistently, there was a general attenuation in the proliferation of total CD3+ and CD4+ T cells after 5-day cocultures with multiple iPSC-EC populations compared to HUVECs. In support of this, there was a lower level of secreted proinflammatory cytokines from iPSC-EC cocultures than that from HUVEC cocultures.

The recent progress in clinical application of iPSC–derived cells demonstrates significant potential for the development of regenerative medicine [32–34]. While avoiding the ethical and allogeneic immune rejection issues associated with human embryonic stem cell–based therapy, there is prospect that the creation of iPSC derivatives from patients would facilitate personalized cell therapy because the immune match would avoid nonself rejection and prevent the need for lifelong immunosuppressive treatment with the associated side effects [6, 35]. However, it has been reported that cells derived from iPSCs can be immunogenic to the autologous immune system possibly due to aberrant gene expression followed by proteome alterations and neoantigen formation [14, 36, 37]. The experimental work in vitro and after transplantation has shown that certain cell types derived from mouse iPSCs, such as cardiomyocytes, but not hepatocytes and neuronal cells, are immunogenic in syngeneic recipients, and other immunogenic cell types such as ECs are immune tolerated [11, 38, 39]. The study by Zhao et al. [14] also revealed that autologous human iPSC–derived smooth muscle cells (SMCs) were highly immunogenic, whereas autologous human iPSC–derived retinal pigment epithelial cells (RPEs) were immune tolerated. This differential immunogenicity was due in part to abnormal expression of immunogenic antigens in human iPSC–derived SMCs, but not in human iPSC–derived RPEs.

Despite many reports on the characterization of microvessel formation of iPSC-ECs, there is a lack of comparable studies examining the expression of MHC molecules, Class I and Class II, at the basal level and in response to inflammatory stimulation, between iPSC-ECs and primary ECs. The immunological significance of MHC Class I and Class II present on the antigen-presenting cells is determined by their communication with CD8+ T cells and CD4+ T cells for immune recognition, respectively [21]. Therefore, the MHC molecules play a vital role in recognition of self and nonself, and these properties are critically important when considering a potential application of iPSC-ECs for cell therapy. By close contacts on circulating T cells, native vascular ECs have been well demonstrated to possess semiprofessional antigen-presenting cell function and recruitment of antigen-specific T cells [22]. The major finding of this study was a lack of MHC Class II expression across multiple iPSC-ECs following IFN-*γ* stimulation in contrary to HUVECs, suggesting that iPSC-ECs may not adopt an activated phenotype for interacting with CD4+ T cells and this may result in a potential disparity in the recruitment of CD4+ T cells between iPSC-ECs and primary ECs. This is the first report, to our knowledge, of the phenomenon MHC phenotype of iPSC-ECs in response to cytokine stimulation compared with primary ECs, and there is no bench marker reference that can be referred to or discussed within the publication domain. Nevertheless, our observation of inflammatory response of HUVECs in relation to MHC expression is consistent to the previous study by Wedgwood, Hatam, and Bonagura [30]. They showed that HUVECs expressed MHC Class II following IFN-*γ* induction, which enable them to present antigens to CD4+ T cells and activate the adhesion of CD4+ T cells to HUVECs. Therefore, the inability of IFN-*γ*-treated iPSC-ECs to function as semiprofessional antigen-presenting cells revealed by this study may compromise their communication with CD4+ T cells for an immune response.

In addition to the MHC molecule recognition, effective activation of immune cells requires stable attachment to ECs. The adhesion molecule E-selectin is expressed on activated but not resting ECs, and it is involved in the initial capture and rolling of leukocytes during the recruitment process of the leukocyte adhesion cascade. Both ICAM-1 and VCAM-1 are required for firm adherence of leukocytes to ECs during the second recruitment step [40]. Consistent to previous reports, our results showed that HUVECs constitutively expressed ICAM-1, but not VCAM-1 and E-selectin, and they responded to TNF-*α* promotion of ICAM-1 expression to a high level and induction of both VCAM-1 and E-selectin. iPSC-ECs generally replicated HUVECs with basal expression of ICAM-1 and further increased expression of ICAM-1 following TNF-*α* treatment. This TNF-*α* upregulation of surface expression of ICAM-1 on iPSC-ECs agrees with previous studies [31, 41, 42]. Interestingly, our data revealed a heterogeneous response to TNF-*α* induction across different types of iPSC-ECs with a differential expression of E-selectin and VCAM-1, which reflects the contradicting data available in published reports [31, 41–43]. Our finding of the negative response of iPSC-ECs to TNF-*α* induction of E-selectin and VCAM-1 supports previous studies showing that human iPSC–derived brain microvascular EC (BMEC)-like cells, iPSC–derived arterial-like ECs and iPSC–derived venous-like ECs also lacked expression of E-selectin and VCAM-1 after the combination treatment of TNF-*α* with IFN-*γ* [42, 43], whereas the positive response is in agreement with other reports, demonstrating that iPSC-ECs were consistently induced by TNF-*α* to express E-selectin and VCAM-1 [31, 41]. The reason for the heterogeneous response to TNF-*α*-induced upregulation of the expression of VCAM-1 and E-selectin among individual iPSC-ECs is not clear, and it is probably due to the differences in the somatic cell origin of iPSCs (N8 and 01434 derived from fibroblast, and 11713 derived from PBMC), differentiation protocols, cell expansion process and culture methodologies. In fact, recent studies showed that by two different well-established protocols, human iPSC–derived BMEC-like cells lacked expression of E-selectin and VCAM-1 after the combination treatment of TNF-*α* with IFN-*γ*, however, with a new differentiation protocol and the extended EC culture method as well as the conditioned culture medium, iPSC–derived BMEC-like cells emerged to exhibit constitutive expression of E-selectin and VCAM-1, and the latter was upregulated by cytokines [43, 44].

The functional importance of our finding is that iPSC-EC cocultures provoked differential dynamics with slowness and weakness of alloreactivity of PBMCs, alongside a general reduction in CD3+ and CD4+ T cell proliferation and decreased inflammatory cytokine responses of PBMCs compared to HUVEC cocultures. This is consistent with observed failure of iPSC-ECs to present MHC Class II molecule, critical for the recruitment of CD4+ T cells, and decreased cell surface expression of TNF-*α* upregulation of both ICAM-1 and VCAM-1, the key adhesion molecules known to be involved in the firm adhesion and interaction of immune cells with ECs during inflammatory cytokine stimulation. In particular, two types of iPSC-ECs, 01434-ECs and 11713-ECs, reduced and delayed significantly the allogenic reactivity and IFN-*γ*-stimulated proliferation of PBMCs. In comparison with HUVECs, the reduced ability of iPSC-ECs to stimulate proliferation of immune cells may also be explained by a potential involvement of other costimulatory markers such as ICOSL and OX40L, as these have also been shown to be involved in activated EC function [45, 46], and there may be varying levels of these molecules in response to inflammatory stimuli between iPSC-ECs and HUVECs. Additionally, N8-ECs behaved differently from other types of iPSC-ECs in eliciting a PBMC allo-response and an inflammatory cytokine response. This difference may be correlated with their differential basal expression and heterogeneous response to cytokine stimulation of adhesion molecules. For example, N8-ECs possessed a more robust constitutive expression level of ICAM-1 and TNF-*α*-induced expression level of VCAM-1 than those seen on 11713-ECs. Differences in somatic cell origin of iPSC lines (N8 and 01434 derived from fibroblast, and 11713 derived from PBMC) may be responsible for some of expressional and functional differences seen between the iPSC-EC populations. Although not within the scope of this study, expansion of the work to include additional iPSC-EC lines would enable this to be investigated further.

In addition to the expression of MHC Class II in response to inflammatory stimuli, HUVECs have multiple mechanisms in which they interact with the immune system to respond to environmental cues. In both N8-EC and 11713-EC, T cell proliferation was observed to be higher than that of the autologous control, indicating that these EC lines were still able to initiate an immune reaction, albeit lower than that of HUVECs, and this was also reflected in the cytokine profiles obtained. However, the 01434-ECs were unable to initiate T cell proliferation above that of the negative control (Neg control) in these assays, although cytokine production (IFN-*γ*, TNF-*α*, and IL-2) was increased in these cocultures, indicating some level of immune response. These data indicate that among the iPSC-EC lines, although a similar phenotype is obtained to resting HUVEC, the ability to recapitulate the activated function of the mature cell type in these assays appears variable. This variability in recapitulating the functions of the mature cell type, may have resulted from variability in their maturation levels in culture, or as a result of variable efficiency of differentiation. Future studies investigating the function of iPSC-EC both at rest and in an activated state will be useful in further understanding these differences between the iPSC differentiated and mature cell type, and how, if any, this may contribute to their functional differences in vivo.

Previous studies have explored the immunogenicity of syngeneic murine iPSCs and their derivatives, including iPSC-ECs. Guha et al. [39] revealed a lack of T cell proliferative response to syngeneic iPSC-ECs in coculture experiments and a lack of T cell infiltration in transplanted iPSC-ECs of syngeneic mice. Another study showed that autologous murine iPSC-ECs elicit an immune response that resembles the one against a native murine aortic ECs, and transplanted iPSC-EC are immunologically accepted in syngeneic murine recipients [11]. The studies suggest that autologous iPSC-ECs would not elicit immune rejection in the host due to self-tolerance. However, human iPSC-ECs have not been comprehensively tested for their immunological activities.

While our findings of immune phenotypes of iPSC-ECs in comparison with primary ECs is interesting, it may be important to note that this in vitro study does not precisely replicate the complexity of immunogenicity which occurs in pathophysiological environment and clinical sittings. Although in vitro investigations provide valuable contributions towards the understanding of immunological processes, it is important to acknowledge that our observation warrants further investigation. It is also worth noting that the lack of significance in some of these assays is likely a reflection of the variability in immune responses observed across different assays, which may be due to many factors including EC growth and general health as well as the responsiveness of donor PBMC, and the timing of end point assays. Particularly interesting is the 11713-EC response, considering the real-time/kinetic data in which PBMC exhibited very low proliferation in response to 11713-EC, but that this proliferation had just started to increase at the end of the 5-day co-culture. The use of Ki67 to assess proliferation in the end point assay may be capturing this increase of proliferation at the end of the assay in 11713-EC compared with N8-EC and 01434-EC cocultures in which kinetic data indicated the PBMC proliferation had slowed. This data further supports the use of kinetic analysis of cell proliferation complementary to the end point analysis.

The variability of surface molecule expression and functional behavior among different iPSC-EC populations shown in the present study may reflect the heterogeneity of vascular ECs. It is important to remember that human vascular ECs are variably immunogenic, depending on tissue source. ECs consisting of arterial, venous, and capillary types are heterogeneous populations in various tissue and organ vascular beds, showing different patterns of gene expression and different contribution to inflammation [19, 47–49]. Indeed, Rufaihah et al. [50] reported that human iPSC-ECs purified based on CD31 expression were heterogeneous comprising of arterial, venous, and lymphatic subtypes and they exhibited functional heterogeneity. Several recent studies have attempted to induce human iPSC-ECs to express tissue-specific functions such as iPSC–derived arterial-like ECs and iPSC–derived venous-like ECs [42, 51, 52]. As the core objective of regenerative medicine for the ischemic vascular disease is to replace dysfunctional or injured ECs and restore vascular function, a comprehensive assessment of immunogenicity profiles of various autologous and allogeneic tissue-specific iPSC-EC types is essential. The identification of these iPSC-ECs with reduced immune responses is important for the successful clinical application of these cell types.

Taken together, this study demonstrates a consistent lack of IFN-*γ*-induced surface expression of MHC Class II molecule among iPSC-EC populations, a reduction in alloreactive proliferation of immune cells and CD4+ T cells, and a weak inflammatory immune response provoked by iPSC-EC populations compared with primary ECs, providing a better understanding of the immune phenotype and immunomodulatory capacity of iPSC-ECs. Our work also indicates that iPSC-ECs may possibly exhibit a low immunogenicity in a transplant setting and facilitate their integration with the host tissue, which has significant implications for the safe and effective use of iPSC-ECs in vascular regenerative medicine or tissue engineering.

## Figures and Tables

**Table 1:** Designed primer pairs for qPCR.

Gene	Accession number	Primer sequence (5′ to 3′)	PCR product (bp)
OCT4	NM_002701	F: GAC AGG GGG AGG GGA GGA GCT AGG	143
		R: CTT CCC TCC AAC CAG TTG CCC CAA AC	

NANOG	NM_024865.2	F: TGC CTC ACA CGG AGA CTG TC	65
		R: AGG GCT GTC CTG AAT AAG CA	

CD31	NM_000442.4	F: GTC CCT GAT GCC GTG GAA A	212
		R: TAA GAA CCG GCA GCT TAG C	

VEGFR2	NM_002253.2	F: CCC TGC GAA GTA CCT TGG TT	218
		R: TCT GGG GTG GGA CAT ACA CA	

VE-cadherin	NM_001795.3	F: CTT CAC CCA GAC CAA GTA C	209
		R: AAT CCA GAG GCT TCATGG G	

vWF	NM_000552.3	F: GCT GAC ACC AGA AAA GTG CC	195
		R: GTC CCC AAT GGA CTC ACA GG	

GAPDH	NM_002046.3	F: ACG AAT TTG GCT ACA GCA ACA GGG	188
		R: TCT ACA TGG CAA CTG TGA GGA GG	

Abbreviations: F, forward primer; qPCR, quantitative PCR; R, reverse primer; VE-cadherin, vascular endothelial-cadherin; VEGFR2, vascular endothelial growth factor receptor 2; vWF, von Willebrand factor.

## Data Availability

All data that support the findings of this study are included within the article and the Supporting Information file.

## Supporting Information

Supporting Information 1 Figure S1: Validation of human primary fibroblasts prior to use of co-culture model of angiogenesis. Videos S1–S2: LDL uptake by HUVECs and iPSC-ECs over the 7-h time course. Videos S3–S6: Scratch wound cell migration of untreated and VEGF-treated HUVECs, and untreated and VEGF-treated iPSC-ECs over the 23-h time course.

Supporting Information 2 Videos S7–S10: Proliferation of PBMCs (donor 1) in co-culture with HUVECs or N8-ECs in the absence or presence of IFN-over a period of 5 days.
